# The Neck Disability Index Reflects Allodynia and Headache Disability but Not Cervical Musculoskeletal Dysfunction in Migraine

**DOI:** 10.1093/ptj/pzac027

**Published:** 2022-03-01

**Authors:** Zhiqi Liang, Lucy Thomas, Gwendolen Jull, Julia Treleaven

**Affiliations:** The University of Queensland, School of Health and Rehabilitation Sciences, St Lucia, Queensland, Australia

**Keywords:** Disability, Migraine, Musculoskeletal, Neck Pain, Sensitization

## Abstract

**Objective:**

The Neck Disability Index (NDI) is a self-rated disability tool originally developed for whiplash-associated disorders and validated in cervical musculoskeletal conditions. It is now commonly used to assess neck disability in migraine, but it is unknown whether NDI scores relate to migraine and hypersensitivity, cervical musculoskeletal dysfunction, or both. This single-blinded observational study aimed to determine whether the presence of cervical musculoskeletal dysfunction, migraine features, and hypersensitivity predict NDI scores and whether alternate versions of the NDI (NDI-physical, NDI-8, NDI-5) relate more to cervical musculoskeletal dysfunction.

**Methods:**

Migraine and neck pain features, the Headache Impact Test (HIT-6), NDI, Allodynia Symptom Checklist (ASC12), and pressure pain thresholds were assessed in 104 participants with migraine and neck pain, 45 previously identified with cervical musculoskeletal dysfunction and 59 without. The NDI score was regressed on the presence or absence of cervical dysfunction, migraine features, HIT-6, total pressure pain threshold, and ASC12 while accounting for neck pain features. The presence of cervical dysfunction was regressed on the scores of NDI versions.

**Results:**

The ASC12 (standardized ß = 0.20) and HIT-6 (standardized ß = 0.18) were significantly predictive of total NDI score, as were neck pain intensity (standardized ß = 0.32) and frequency (standardized ß = 0.44). No scores from alternate NDI versions related to cervical dysfunction.

**Conclusion:**

The NDI score is a complex measure of neck disability influenced by migraine disability and hypersensitivity beyond the presence of cervical musculoskeletal dysfunction. This has implications for the clinical interpretation of NDI scores in patients with migraine.

**Impact:**

Many patients with migraine and neck pain report neck disability; therefore, it is important to understand if migraine impacts neck disability. The results of this study indicate that clinicians need to consider migraine-related disability and hypersensitivity when managing neck disability in this population.

## Introduction

Approximately 80% of individuals with migraine also experience neck pain,^1^ which compounds their physical burden.^2^ It is therefore important to assess disability related to neck pain in this population. The Neck Disability Index (NDI)^3^ is a self-rated disability tool implemented on patients with cervical musculoskeletal disorders, but it is increasingly used to assess neck pain disability in migraine.^4–7^ Originally designed for whiplash-associated disorders,^3^ the NDI has since been validated in various cervical musculoskeletal disorders.^8^ However, the NDI is yet to be validated in migraine, which can have different underlying neck pain mechanisms from cervical disorders. Benign lesions in cervical musculoskeletal structures are local nociceptive sources in cervical disorders and present clinically with a group of cervical musculoskeletal impairments.^9^^,^^10^ On the other hand, neck pain in migraine could, either in part or full, stem from a coexisting cervical musculoskeletal disorder or be part of the migraine symptomology because of the convergence of afferent neurons from trigeminal and cervical regions within the trigeminocervical nucleus.^11^ Furthermore, bidirectional sensitization in this region could also result in migraine-associated hypersensitivity of the neck^12^ contributing to the neck disability. It is therefore possible that besides cervical musculoskeletal dysfunction, migraine features and hypersensitivity may also explain neck disability in migraine. This has important implications for the clinical interpretation of NDI scores in migraine.

There are other indications that NDI scores may be influenced by migraine factors. Some NDI items, such as headache intensity and frequency, sleep quality, and the ability to concentrate, may be attributable to the migraine itself and be difficult to dissociate from neck pain. Excluding such items may provide a more accurate assessment of neck physical disability in migraine. Previous work evaluating the measurement properties of the NDI may provide some direction. A factor analysis conducted on data from a population with neck pain disorders confirmed that the NDI mostly measures 1 construct, that is, neck disability. However, the researchers proposed separating the 10 items into 2 subconstructs of physical and mental functions (NDI-physical: personal care, lifting, work, driving, sleeping, and recreation; NDI-mental: neck pain, reading, headaches, and concentration).^13^ On the other hand, Rasch analyses of NDI data^14^^,^^15^ suggested that measurement unidimensionality would be better achieved if some items were removed to leave either 8 (NDI-8)^14^ or 5 (NDI-5)^15^ items (ie, for NDI-8, remove headache and lifting; for NDI-5, remove headache, lifting, pain intensity, sleeping, and reading). It is possible that the NDI-physical, NDI-8, and/or NDI-5 would better reflect neck physical disability than the total original 10-item NDI.

The first aim of this study was to examine if the presence of cervical musculoskeletal dysfunction, migraine features, disability, and hypersensitivity could predict the total scores of the NDI. We hypothesized that all variables would be associated with NDI scores, even after accounting for neck pain history, intensity and frequency. The second aim was to determine if alternate versions of the NDI were reflective of cervical physical limitations by testing their association with the presence of cervical musculoskeletal dysfunction. The hypothesis was that NDI versions would be associated with cervical dysfunction.

## Methods

This study is a secondary analysis of a cross-sectional study examining cervical musculoskeletal dysfunction in individuals with migraine (n = 124) compared with healthy controls (n = 32) and individuals with idiopathic neck pain (n = 21). Details of the methodology are provided elsewhere.^16^ In brief, adults aged 18 to 65 years were recruited in each category from the community between December 2018 and March 2020. Individuals with migraine were included if their main headache was diagnosed as either episodic or chronic migraine according to the International Classification of Headache Disorders^17^ criteria. The presence of a second headache was permissible provided it was a migraine-like or tension-like headache but not permissible if it was cervicogenic.^17–19^ Exclusion criteria included a diagnosis of specific neck disorders, cervical surgery, or systemic diseases affecting the cervical spine. Ethical approval for the study was gained from the University of Queensland Human Research Ethics Committee (2018001128), and all participants provided written consent to participate.

Participants enrolled in the study provided information on features of their migraine and neck pain over the past 3 months (length of history, frequency, average pain intensity). They completed questionnaires on neck disability (NDI),^3^ headache disability (Headache Impact Test [HIT-6],^20^ and allodynia (Allodynia Symptom Checklist [ASC12]).^21^ All participants underwent clinical tests, which were undertaken by an assessor who was blinded to the participants’ group status. Tests included assessments of cervical musculoskeletal function (range of motion, movement accuracy, manual examination of segmental joint pain and dysfunction, the craniocervical flexion test, flexor and extensor strength tests, joint position sense) and pressure pain thresholds (PPTs).^16^

### Current Study

The data of interest for this study were NDI and HIT-6 scores, migraine and neck pain features (history, frequency, and average intensity), measures of pain hypersensitivity (PPTs and ASC12 scores), and the participant’s cervical musculoskeletal function or dysfunction status. Only participants with migraine and neck pain were included in this current study (n = 110).

### Neck Disability Index (NDI)

Participants were instructed to select the statement that best applied to how their neck pain affected their ability to manage each of the 10 items on the NDI. Each item is scored from 0 to 5, giving a maximal total score of 50. The item on driving was sometimes omitted if not applicable to the participant, resulting in a maximal score of 45. Hence, the total NDI score was calculated as a percentage of 50 or 45 points. For the other NDI versions, the 6 items deemed to measure physical function (NDI-physical) included personal care, lifting, work, driving, sleeping, and recreation, whereas the remaining 4 items (neck pain, reading, headaches, and concentration) constituted measures of mental function (NDI-mental).^13^ NDI-8^14^ excluded the items on lifting and headaches, whereas NDI-5^15^ consisted only of the items relating to personal care, concentration, work, driving, and recreation. As with the total NDI score, the percentage scores for the total NDI, NDI-physical, NDI-mental, NDI-8, and NDI-5 were used in analyses.

### Migraine and Neck Pain Features

Participants completed a questionnaire to provide information from which the following data were extracted. History of migraine was the number of years since diagnosis. Headache frequency was the average number of headache days per month over the past 3 months. Average migraine intensity was rated on a scale from 0 to 10. Similarly, history of neck pain was the number of years since first experiencing neck pain. Average neck pain was rated on a scale from 0 to 10. Neck pain frequency was rated in the following categories: monthly, fortnightly, weekly, and daily or almost daily.

### Pressure Pain Thresholds

PPTs were obtained unilaterally using a digital algometer with a probe size of 1 cm^2^ (Somedic Production AB, Stockholm, Sweden) on the more symptomatic side at the forehead, neck (C6 articular pillar), and tibialis anterior muscle belly (remote site). The side of testing was randomized if the participant had equal symptoms bilaterally. Three measures were obtained for each location, with 30-second intervals between PPT trials.^9^ Mean values for each site were calculated and then summed to give the total PPT for use in analyses.

### Cervical Musculoskeletal Status

The cervical musculoskeletal status of each migraine participant was taken from the results of our previous study. The presence of cervical musculoskeletal function or dysfunction in each participant with migraine was determined using cluster analysis, which grouped each participant with migraine with the healthy controls or with individuals with idiopathic neck pain. These clusters were based on each individual’s combined outcomes across the cervical musculoskeletal assessments.^16^ Only 2 clusters were formed: all of the healthy controls were grouped into 1 cluster, and all of the individuals with idiopathic neck pain were grouped into the other cluster. Participants who had migraine and were grouped into the same cluster as individuals with idiopathic neck pain were considered to have cervical dysfunction, whereas those who were grouped into the same cluster as healthy controls were considered to have no cervical dysfunction.

### Data Analyses

Data were inspected and tested using the Shapiro–Wilk test for normality. Only participants without missing data were included in the analyses. Simultaneous multiple linear regression was chosen to examine the relative extents of influence from all conceivable variables that may contribute to the total NDI score. The variables of interest to the research question were migraine features (history, intensity, and frequency), HIT-6, total PPT, ASC12, and presence or absence of cervical musculoskeletal dysfunction. Neck pain features (history, intensity, and frequency) were also accounted for in the model, because these have a direct impact on the experience of neck pain. Relationships between all independent variables and total NDI score were tested individually to satisfy the assumption for linearity before inclusion in the model. To ensure all assumptions for the regression model were met, multicollinearity statistics and tests for independence of residuals were performed. Normality and constant variance of residuals were also confirmed through visual inspection of residual quantile-quantile plots and scatterplots of standardized residuals versus standardized predicted values.

For the second aim, univariate binary logistic regression was used to determine whether NDI-physical, NDI-mental, NDI-8, and/or NDI-5 scores were individually associated with the presence of cervical musculoskeletal dysfunction. The Box-Tidwell test was used to confirm the assumption of linearity between each predictor and logit. Two-tailed *P* values were calculated with the significance level set at .05. Analyses were performed using SPSS software, version 26 (IBM, Armonk, NY, USA). Because this was part of a larger study, a priori sample size calculation was not indicated. Derived data supporting the findings of this study are available from the corresponding author on request.

### Role of the Funding Source

The funders played no role in the design, conduct, or reporting of this study.

## Results

In the original study, 110 of the 124 individuals with migraine reported neck pain and were eligible for this study. Six did not complete the NDI and were excluded. Of the 104 individuals retained for analysis, 87 were classified as having frequent episodic migraine and 17 were classified as having chronic migraine. Forty-five were grouped with participants who had idiopathic neck pain and presented with cervical musculoskeletal dysfunction, and 59 were grouped with healthy controls and were therefore regarded as having no relevant cervical musculoskeletal dysfunction. Participant characteristics are detailed in Table 1. A further participant did not answer the question on the history of neck pain and was excluded from the first analysis.

**Table 1:** Participant Characteristics^a^

Characteristic	Mean	95% CI		No. (%) of Participants
		Lower	Upper	
Age, y	41.0	38.8	43.1	
Migraine history, y	19.1	16.9	21.4	
Average migraine intensity, 0–10	6.7	6.4	7.0	
Headache frequency, d/mo	7.9	6.7	9.1	
Neck pain history, y*^b^*	12.4	10.6	14.2	
Average neck pain intensity, 0–10	4.6	4.3	4.9	
HIT-6 score/78	63.9	62.9	64.8	
ASC12 score/24	5.0	4.5	5.7	
Total PPT, kPa	745.2	700.9	789.5	
Total NDI, %	25.0	22.8	27.1	
NDI-physical, %	19.3	17.1	21.6	
NDI-mental, %	33.3	30.6	36.0	
NDI-8, %	22.3	20.1	24.5	
NDI-5, %	18.5	16.2	20.7	
Neck pain frequency				
Monthly				10 (9.6)
Fortnightly				14 (13.5)
Weekly				37 (35.6)
Daily or almost daily				43 (41.3)
No. of women				92 (88.5)
Cervical MSK dysfunction, present				45 (43.3)

^*^a^*^There were 104 participants unless otherwise indicated. ASC12 = Allodynia Symptom Checklist; HIT-6 = Headache Impact Test; MSK = musculoskeletal; NDI = Neck Disability Index; NDI-5 = NDI items personal care, concentration, work, driving, and recreation; NDI-8 = all items on the NDI except for lifting and headaches; NDI-mental = NDI items neck pain, reading, headaches, and concentration; NDI-physical = NDI items personal care, lifting, work, driving, sleeping, and recreation; total NDI = total percentage score on the NDI; PPT = pressure pain threshold.

^*^b^*^There were 103 participants.

### Predictors of Total NDI Score (N = 103)

The overall model derived from the multiple linear regression was significantly predictive of total NDI score (*R*^2^ = 0.45; *F*_10,92_ = 7.6; *P* < .001). As reported in Table 2, the significant contributors to the model were neck pain frequency (β = 5.08; *P* < .001), neck pain intensity (β = 2.26; *P* < .001), ASC12 (β = 0.71; *P* = .018), and HIT-6 (β = 0.42; *P* = .049). The remaining predictors, including history of neck pain, migraine features, presence of cervical dysfunction, and total PPT, did not contribute significantly to NDI scores. All assumptions for the regression model were met (collinearity statistics of at least 0.68, variance inflation factor all <2.0, Durbin-Watson statistic = 1.96, no obvious signs of funneling on scatterplots of standardized residuals vs standardized predicted values, no obvious deviations on quantile-quantile plots, and no influential cases biasing the model [Cook distance of <1 for all participants]).

**Table 2:** Coefficient Values of Predictors Included in the Regression Model for the Total Percentage Score on the Neck Disability Index^a^

**Predictor**	**β (95% CI)**	**β SE**	**Standardized ß**	***P***
Constant	**−29.74 (−54.58 to − 4.91)**	**12.51**		**.019**
Neck pain frequency	**5.08 (3.17 to 6.99)**	**0.96**	**0.44**	**<.0001**
Neck pain intensity	**2.26 (1.03 to 3.50)**	**0.62**	**0.32**	**<.0001**
ASC12 score	**0.71 (0.12 to 1.30)**	**0.30**	**0.20**	**.018**
HIT-6 score	**0.42 (0.00 to 0.84)**	**0.21**	**0.18**	**.049**
Neck pain history	−0.08 (−0.30 to 0.14)	0.11	-0.07	.45
Migraine history	0.02 (−0.15 to 0.19)	0.09	0.02	.82
Migraine intensity	−0.17 (−1.58 to 1.24)	0.71	-0.02	.81
Migraine frequency	0.11 (−0.19 to 0.41)	0.15	0.06	.47
PPT total	0.00 (−0.01 to 0.01)	0.00	0.05	.58
Cervical MSK dysfunction	−1.64 (−5.33 to 2.05)	1.86	-0.07	.38

^*^a^*^Bold type indicates factors that contributed to the model. ASC12 = Allodynia Symptom Checklist; HIT-6 = Headache Impact Test; MSK = musculoskeletal; PPT = pressure pain threshold.

### Associations Between Cervical Musculoskeletal Dysfunction and Scores From NDI Versions (NDI-Physical, NDI-Mental, NDI-8, and NDI-5) (N = 104)

Table 3 presents the NDI, NDI-physical, NDI-mental, NDI-8, and NDI-5 scores as well as individual item scores of participants with migraine, with and without cervical musculoskeletal dysfunction. The results of the univariate binary logistic regressions detailed in Table 4 showed that no variation of the NDI was significantly predictive of cervical musculoskeletal dysfunction. All assumptions for the regression analyses were met.

**Table 3:** NDI Scores in Participants With Migraine, With and Without Cervical Dysfunction^a^

**NDI**	**Mean (95% CI) for Participants**	
	**Without Cervical Dysfunction (n = 59)**	**With Cervical Dysfunction (n = 45)**
Total, %	25.3 (22.0 to 28.6)	24.5 (21.8 to 27.3)
NDI-physical, %	19.5 (16.3 to 22.8)	19.1 (16.0 to 22.2)
NDI-mental, %	33.9 (29.9 to 37.9)	32.6 (29.1 to 36.0)
NDI-8, %	22.4 (19.1 to 25.7)	22.2 (19.3 to 25.0)
NDI-5, %	19.0 (15.6 to 22.3)	17.8 (14.9 to 20.7)
Individual NDI items (0–5)		
Item 1: pain intensity	1.4 (1.2 to 1.6)	1.4 (1.2 to 1.7)
Item 2: personal care	0.2 (0.1 to 0.4)	0.1 (0.0 to 0.3)
Item 3: lifting	0.9 (0.6 to 1.2)	0.8 (0.5 to 1.1)
Item 4: reading	1.4 (1.1 to 1.6)	1.5 (1.2 to 1.7)
Item 5: headaches	2.8 (2.4 to 3.1)	2.6 (2.2 to 2.9)
Item 6: concentration	1.2 (1.0 to 1.5)	1.0 (0.8 to 1.3)
Item 7: work	1.2 (0.9 to 1.5)	0.9 (0.6 to 1.1)
Item 8: driving	0.9 (0.7 to 1.2)*^b^*	1.2 (1.0 to 1.5)*^c^*
Item 9: sleeping	1.4 (1.2 to 1.7)	1.5 (1.2 to 1.8)
Item 10: recreation	1.2 (1.0 to1.4)	1.2 (1.0 to 1.4)

*^a^* NDI = Neck Disability Index; NDI-5 = NDI items personal care, concentration, work, driving, and recreation; NDI-8 = all items on the NDI except for lifting and headaches; NDI-mental = NDI items neck pain, reading, headaches, and concentration; NDI-physical = NDI items personal care, lifting, work, driving, sleeping, and recreation; total NDI = total percentage score on the NDI.

^*^b^*^n = 58.

^*^c^*^n = 40.

**Table 4:** Results of Univariate Binary Logistic Regression of Cervical Musculoskeletal Function or Dysfunction on the NDI^a^

**NDI**	**Overall Model**		**Predictor**	**β**	**β SE**	**Wald Statistic**	***df***	***P***	**Exp β**	**95% CI for Exp β**
	**χ** ^**2**^_**1**_	***P***								
NDI-physical	0.038	.846	NDI-physical	--0.003	0.017	0.038	1	.85	0.997	0.963 to 1.031
			Constant	--0.206	0.387	0.283	1	.59	0.814	
NDI-mental	0.246	.620	NDI-mental	--0.007	0.015	0.245	1	.62	0.993	0.965 to 1.021
			Constant	--0.033	0.520	0.004	1	.95	0.968	
NDI-8	0.010	.920	NDI-8	--0.002	0.018	0.010	1	.92	0.998	0.964 to 1.033
			Constant	--0.231	0.439	0.278	1	.60	0.793	
NDI-5	0.274	.600	NDI-5	--0.009	0.017	0.272	1	.60	0.991	0.958 to 1.025
			Constant	--0.104	0.375	0.077	1	.78	0.901	

^*^a^*^NDI = Neck Disability Index; NDI-5 = NDI items personal care, concentration, work, driving, and recreation; NDI-8 = all items on the NDI except for lifting and headaches; NDI-mental = NDI items neck pain, reading, headaches, and concentration; NDI-physical = NDI items personal care, lifting, work, driving, sleeping, and recreation.

## Discussion

The first hypothesis of this study was partially upheld. The NDI score in migraine was significantly influenced by neck pain frequency and intensity, but it was also influenced by allodynia (ASC12) and headache disability (HIT-6). Unexpectedly, the NDI score was not related to physical limitations of the cervical spine (ie, musculoskeletal dysfunction). Likewise, and contrary to our second hypothesis, no alternate version of the NDI was associated with physical limitations of the cervical spine in participants with migraine and neck pain. These findings indicate that reported neck disability in migraine is more closely linked to migraine pathophysiology, and this has important implications for the use and interpretation of the NDI in future research on migraine-associated neck pain. The major strength of the study is that it is the first, to our knowledge, to investigate the influence of migraine-related factors on reported neck disability, taking into account all known possible contributors to neck disability in migraine.

Some studies suggest that self-reported neck pain in participants with migraine is associated with factors such as cutaneous allodynia, neck mobility, and muscle tenderness or function,^22–24^ but our study only found an association between self-reported neck disability and allodynia. This may reflect our attention in interpreting test results with respect to deficits in overall musculoskeletal function rather than individual impairments or pain sensitivity. Neck pain frequency and intensity were, not surprisingly, significant predictors of the NDI score because they relate directly to the experience of neck pain. However, these variables are of limited value in understanding the underlying cause of the neck disability reported, because neck pain can be due to either local cervical nociception or migraine sensitization, or both.^11^ More importantly, the ASC12 and HIT-6 scores were also significant predictors of the NDI score, whereas the presence of cervical musculoskeletal dysfunction was not. This suggests that the neck disability reported by individuals with migraine is influenced by migraine-related factors of allodynia and headache disability, more so than physical limitations of the neck. Previous studies have also noted relationships between NDI scores and variables related to migraine, such as PPT,^25^ muscle tenderness in the head and neck,^22^ and headache frequency.^4^^,^^5^ However, PPT and headache frequency were not significant contributors to NDI score in our study when the other variables were considered.

Previous studies have looked to modify the NDI by removing items to create a more specific measure of neck disability in cervical musculoskeletal pain states with the resultant creation of the NDI-8^14^ and NDI-5.^15^ There has also been a recommendation to split the NDI into NDI-physical and NDI-mental.^13^ However, we found that no version had significant association with cervical musculoskeletal dysfunction in migraine. This further supports the implication from the first set of findings that neck disability in migraine may be more related to migraine. Just as ictal neck pain can be improved by acute migraine medication,^26^ appropriate management of migraine may be of priority in some participants who have migraine and are seeking to improve neck disability.

The findings of this study indicate that neck disability in migraine, as measured by the NDI, may be more related to migraine factors than local neck dysfunction. This may be due to the nature of neck disability in migraine and/or the measurement properties of the NDI in this population. We specifically asked participants to focus on their neck pain when answering the questions on the NDI. However, participants with migraine often experience neck pain around the time of the migraine episode,^27^^,^^28^ so it is probable that the NDI items include functions that may be difficult for participants to dissociate their neck pain from their migraine, for instance, sleeping, concentration, work, and recreation. Thus, although the NDI gives a measure of neck disability in migraine, the inability to perform the functions can as likely be from the pain and sensitivity associated with the migraine as from nociception and physical limitations of the cervical spine.

### Limitations

The data on migraine and neck pain frequency and intensity are subject to some reporting or recall bias because information sought from participants was the average over the past 3 months to account for the fluctuation of symptoms that typically occur in this population. No power calculations were performed for sample size, but this study presents a secondary analysis.

Neck pain often accompanies migraine, and the NDI is commonly used to assess the neck-related disability. The findings of this study indicate that awareness of possible influences from migraine-related factors is required with the interpretation and use of NDI as an outcome measure in migraine. NDI scores do not necessarily reflect cervical dysfunction and are probably more reflective of migraine mechanisms and disability. Because neck pain is a common complaint in migraine, a valid measure to assess neck-related disability is needed. However, the underlying mechanisms and disability surrounding neck pain and migraine may be highly intertwined and impossible to separate. Further steps towards validation of the NDI may be necessary for this population.
